# Time to Prostate-Specific Antigen Failure as a Unique Prognosticator of Overall Survival in Biochemically Recurrent Prostate Cancer Patients Undergoing Radical Prostatectomy

**DOI:** 10.1155/aiu/2961319

**Published:** 2025-08-28

**Authors:** Tomoyuki Shimabukuro, Takanori Tokunaga, Kosuke Shimizu, Nakanori Fujii, Keita Kobayashi, Toshiya Hiroyoshi, Hiroshi Hirata, Koji Shiraishi

**Affiliations:** ^1^Department of Urology, Graduate School of Medicine, Yamaguchi University, Ube, Yamaguchi, Japan; ^2^Department of Urology, Ube Central Hospital, Ube, Yamaguchi, Japan

**Keywords:** biochemically recurrent prostate cancer, overall survival, prognosticator, radical prostatectomy, time to biochemical recurrence

## Abstract

**Background:** In biochemically recurrent prostate cancer (BRPC), no definitive independent prognostic factors were reported. This study aimed to identify the factors impacting overall survival (OS) in patients with BRPC after radical prostatectomy (RP).

**Methods:** Among 610 consecutive patients who underwent RP between January 2000 and December 2019, with follow-up through December 2024, 152 (25%) patients who developed BRPC were analyzed. The primary endpoint was to identify an independent prognosticator of OS, while the secondary endpoint was to investigate clinical and tumor characteristics in BRPC patients.

**Results:** The median age of the cohort was 67 years. Of the BRPC patients, 37 (24.4%) were managed with observation alone, 80 (52.6%) underwent external beam radiation therapy with or followed by androgen deprivation therapy (ADT), and 35 (23.0%) received ADT alone. During follow-up, two cases of local recurrence and nine cases of distant metastases were observed, with seven patients (1.2%) progressing to castration-resistant prostate cancer. Over a median follow-up of 118 months, 21 all-cause and 5 cancer-specific deaths were recorded. Multivariable analysis identified time to biochemical recurrence (TTBR) as the sole independent significant prognostic factor for OS (hazard ratio: 0.956, 95% confidence interval: 0.916–0.997, *p*=0.036). Kaplan–Meier survival curves, using a TTBR cutoff of 12 months, revealed significant differences in OS between the shorter and longer TTBR cohorts.

**Conclusions:** This long-term retrospective study demonstrates that TTBR may serve as a unique independent prognostic factor for OS in BRPC patients. A TTBR of ≤ 12 months was significantly associated with worse OS, irrespective of clinicopathological risk features.

## 1. Introduction

Radical prostatectomy (RP) remains one of the most widely utilized treatment modalities for localized or locally advanced prostate cancer (PCa). Despite advancements in diagnostic methods and surgical techniques, including the adoption of robot-assisted RP (RARP), a significant proportion of patients experience prostate-specific antigen (PSA) recurrence within 10 years of surgery. Specifically, approximately 20% of men develop PSA recurrence following open radical retropubic prostatectomy (ORRP) [1], while 12%–20% do so after standard RARP [2, 3].

It is well-established that not all men with detectable PSA after surgery will progress to clinical failure, defined as the development of metastases or death from PCa. In fact, it is common to observe cases where PSA levels plateau after prostatectomy without subsequent elevation. Spahn et al. reported in their cohort of 622 patients with intermediate- and high-risk disease that only 52% experienced clinical failure at median times of 9.9 and 5.6 years, respectively. These failures included local recurrence (14%), lymph node metastases (9%), and distant metastases (DMs) (24%) [4].

Several hypotheses have been proposed to explain why detectable PSA levels do not always indicate clinically significant disease. First, residual benign tissue in the prostate bed may produce PSA. Second, PSA can be generated at low levels by nonprostatic cells. Third, PSA elevation may result from residual low-grade PCa destined to follow an indolent course [5, 6]. To clarify the true clinical significance of PSA progression, a thorough evaluation of treatment outcomes is crucial. By analyzing our institutional experience with biochemical recurrence (BCR) following RP, we aim to generate valuable insights into its incidence, associated perioperative clinical and pathological characteristics, treatment sequences, progression to castration-resistant disease, and potential adverse effects on overall survival (OS). This comprehensive analysis seeks to refine our understanding of PSA progression's role in clinical decision-making, particularly in guiding the use of adjuvant treatments.

Balancing treatment efficacy with the risk of overtreatment, quality-of-life deterioration, and financial burden remains a significant challenge. Drawing on 25 years of institutional experience, our study aims to contribute to evidence-based medical practice and support informed decision-making in routine clinical settings.

## 2. Methods

### 2.1. Study Patients

Between January 2000 and December 2019, a total of 1278 patients pathologically diagnosed with PCa were identified at Yamaguchi University Hospital and Ube Central Hospital. Among these, 610 consecutive patients underwent RP. From this cohort, we analyzed 152 patients who subsequently developed biochemically recurrent prostate cancer (BRPC) and had follow-up data available through December 2024. Of these, 87 patients underwent ORRP, while 65 underwent RARP. The primary endpoint was to identify independent prognosticators for OS, while the secondary endpoint was to investigate the clinical and tumor characteristics of BRPC.

### 2.2. Diagnostic and Treatment Procedures

Our eligibility criteria, diagnostic and staging procedures, and pathological diagnoses of RP were reported elsewhere [3].

### 2.3. Data Collection

Clinical data were extracted from written or electronic medical records and, where necessary, supplemented by direct interviews. All data were reviewed using a standardized form by two independent researchers to ensure consistency and accuracy. Discrepancies were resolved by consensus. Eligible patients had a minimum follow-up period of 5 years, with data collection concluding in December 2024.

### 2.4. Study Approval

This study was approved by the Institutional Review Board of the Yamaguchi University School of Medicine (approval number: H2020-229) and the Ube Central Hospital (approval number: 70160502).

### 2.5. Statistical Analysis

Statistical analyses were performed using StatView Version 4.5 (SAS, Cary, NC, USA). Associations between surgical modalities and clinicopathological factors were evaluated using the Fisher's exact probability test for categorical variables and the Mann–Whitney *U* test for continuous variables. OS rates were estimated using the Kaplan–Meier method, and comparisons were conducted using the log-rank test. Univariable and multivariable analyses for predicting OS were performed using the Cox proportional hazards model. All variables in univariable analysis were entered into the multivariable Cox regression model.

A two-sided *p* value of < 0.05 was considered statistically significant.

## 3. Results

### 3.1. Preoperative, Postoperative, and Follow-Up Characteristics

Table 1 summarizes the perioperative and follow-up characteristics of the 152 enrolled patients, categorized by surgical modality. The median age (interquartile range) was 68 years (61–70) for the ORRP group and 68 years (63–70) for the RARP group. Age distribution and D'Amico risk groups were comparable between the two groups.

Significant differences were observed in baseline PSA levels, clinical T stages, and biopsy Gleason scores between the two groups. Baseline PSA levels and clinical T stages were higher in the ORRP group, while biopsy Gleason scores were higher in the RARP group. In terms of prostatectomy specimen pathology, significant differences were found in Gleason scores and surgical margin status, both of which were higher in the RARP group.

The median follow-up duration was 173 months (111–209) for the ORRP group and 83 months (75–108) for the RARP group. BCR occurred in 57% of ORRP patients, with a median time to BCR of 37 months, and in 43% of RARP patients, with a median time to BCR of 20 months. During follow-up, there were 19 all-cause deaths (22%) and 5 PCa-specific deaths (6%) in the ORRP group. In contrast, the RARP group had 2 all-cause deaths (3%) and no PCa-specific deaths.

### 3.2. Local Recurrence and Sites of DMs

Table 2 details local recurrence and sites of DMs. DMs occurred in 7% of ORRP patients, with a median time to DM of 54 months, and in 5% of RARP patients, with a median time to DM of 12 months.

### 3.3. Treatment Sequences After RP

**Figure 1:**
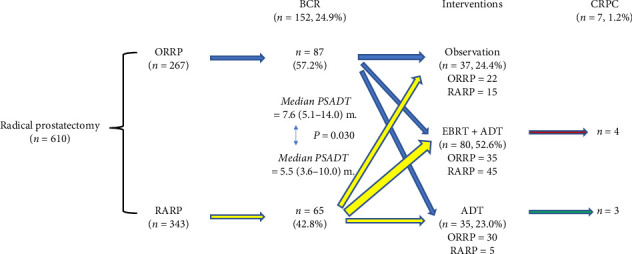
Treatment sequences after radical prostatectomy. Abbreviations: ORRP: open radical retropubic prostatectomy; RARP: robot-assisted radical prostatectomy; BCR: biochemical recurrence; PSADT: PSA doubling time; EBRT: external beam radiation therapy; ADT: androgen deprivation therapy; CRPC: castration-resistant prostate cancer; m: months.

Figure 1 illustrates the treatment sequences during the median follow-up period of 98 months for the 610 patients who underwent ORRP or RARP. Of the 152 patients with BRPC, 37 (24.4%) were managed with observation alone, 80 (52.6%) received external beam radiation therapy (EBRT) combined with androgen deprivation therapy (ADT) or followed by ADT, and 35 (23.0%) were treated with ADT alone. During a median follow-up of 9.8 years, 7 patients (1.2%) progressed to castration-resistant prostate cancer (CRPC).

### 3.4. Investigation for Prognosticators of OS

To identify prognosticators for OS, Cox regression analysis was performed.  Table 3 summarizes the risk factors associated with overall mortality. In the univariable analysis, clinical T4 stage, pathological N1 stage, PSA doubling time at BCR, and time to BCR (TTBR) were significantly associated with shorter OS. In the multivariable analysis, TTBR was identified as the only independent significant prognostic factor for OS.

### 3.5. OS in Two Categorized Cohorts

**Figure 2:**
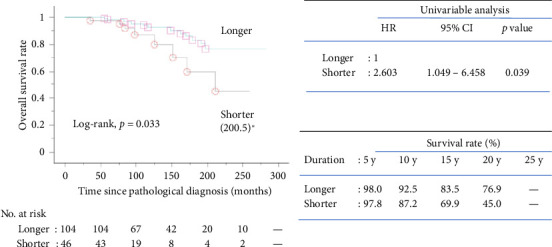
Kaplan–Meier overall survival rate plots of biochemically recurrent prostate cancer patients undergoing radical prostatectomy stratified by time to biochemical recurrence. Abbreviations: shorter: time to biochemical recurrence ≤ 12 months; longer: time to biochemical recurrence > 12 months; HR: hazard ratio; 95% CI: 95% confidence interval; y: years. ^∗^Median survival time (months).

Based on the result of the multivariable analysis, patients were divided into two cohorts according to their TTBR: The shorter cohort (TTBR ≤ 12 months) and the longer cohort (TTBR > 12 months). The Kaplan–Meier survival curves for OS in the two cohorts are presented in Figure 2. The analysis showed a significant difference in OS between the cohorts, with a hazard ratio (HR) of 2.603 (95% confidence interval [CI]: 1.049–6.458, *p*=0.039) for the shorter cohort. The 10-year OS rates were 93% for the longer cohort and 87% for the shorter cohort.

## 4. Discussion

The notable difference in median follow-up duration between ORRP and RARP groups mainly reflects the starting points of introducing these surgical methods in our institution (ORRP from 2000 and RARP from 2012) (Table 1). However, this introduces a potential follow-up bias. We attempted to mitigate this through censoring strategies, hence further prospective validation is warranted.

A systematic review and meta-analysis have shown that BCR is associated with an increased risk of DMs, PCa-specific mortality, and overall mortality. However, these risks are primarily confined to patients with a short PSA doubling time and high Gleason score after RP [7]. In this study, visceral metastases occurred only in the ORRP group (Table 2), which may reflect differences in the surgical era (RARP started in our institution from September 2012), risk stratification, or imaging sensitivity.

Boorjian et al. analyzed 2426 patients with BCR (17% of 14,631 cases) and reported that the time from ORRP to BCR was not significantly associated with the risk of systemic progression or cancer-specific mortality [8]. However, their study defined BCR as PSA ≥ 0.4 ng/mL, included varied utilization of salvage therapies, and did not account for patients treated with RARP. Given the increasing prevalence of RARP, reevaluating the natural history of BRPC patients in the contemporary era is essential. While our analysis focused on OS, our findings support that early biochemical failure portends poor outcomes across multiple endpoints.

In our analysis, a shorter TTBR was significantly associated with worse OS in multivariable analysis adjusted for clinicopathological covariates. However, it cannot be concluded as an independent prognostic factor without broader validation. In addition, it is noteworthy that the high HR for PSA 10.1–20.0 ng/mL and pN1 status may be affected by small sample sizes and low event counts in these strata. Therefore, these findings should be interpreted with caution. Nevertheless, this suggests the critical role of TTBR as the most important risk factor in patients undergoing RP for PCa. These findings hold significant implications for guiding salvage therapies and tailoring postoperative monitoring strategies.

For patients with a shorter TTBR (≤ 12 months), prompt initiation of salvage therapy should be prioritized. Key considerations for these treatments include the following:

1. Salvage radiation therapy (SRT): It should be discussed with patients who have nonmetastatic PCa.
2. ADT: This should be considered as an adjunct to SRT to enhance treatment efficacy.
3. Androgen receptor signaling inhibitors: Agents such as enzalutamide may provide additional therapeutic benefits [9].
4. Advanced imaging techniques: Tools such as multiparametric magnetic resonance imaging (mpMRI) and prostate-specific membrane antigen (PSMA) positron-emission-tomography (PET) scans are valuable for detecting local recurrence post-RP.

**Figure 3:**
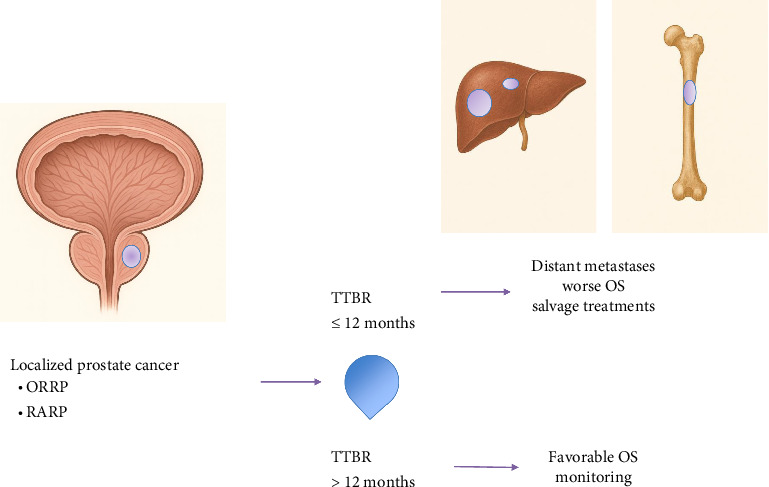
Graphical abstract. Time to biochemical recurrence (TTBR) after radical prostatectomy predicts oncologic outcomes and informs treatment planning. Abbreviations: ORRP: open radical retropubic prostatectomy; RARP: robot-assisted radical prostatectomy; TTBR: time to biochemical recurrence; OS: overall survival.

For patients with a longer TTBR (> 12 months), multivariable analysis revealed a statistically significant association between higher baseline PSA levels, pathological N1 stage, and higher Gleason score and OS (Supporting Table [available here]). These findings highlight the heterogeneity of outcomes among patients with BCR and emphasize that not all patients require aggressive interventions. Nevertheless, vigilant monitoring remains critical to identify disease progression. As a convenience for readers, a graphical abstract was attached (Figure 3).

This study has several limitations. First, the retrospective design and relatively small sample size may limit the generalizability of the findings. Second, variability among surgeons and the individualized use of adjunctive therapies, such as adjuvant and salvage treatments determined by treating physicians, could have influenced outcomes. Finally, we were unable to evaluate PCa-specific survival rates due to the limited number of PCa-specific deaths in our cohort.

## 5. Conclusions

Our extended follow-up study underscores the importance of TTBR as a prognostic factor for OS in patients with BCR following RP for PCa. The identification of shorter TTBR as a significant predictor of poor OS highlights the need for tailored treatment strategies in this subset of patients. Future randomized controlled trials are warranted to clarify the prognostic impact of shorter TTBR and guide evidence-based management strategies on PCa-specific mortality and evidence-based management.

## Figures and Tables

**Table 1:** Baseline and clinicopathological characteristics stratified by surgical group.

	All	ORRP	RARP	*p* value
	(*n* = 152)	(*n* = 87)	(*n* = 65)	
*Preoperative Variables*				
Age at pathological diagnosis (y)				0.975
< 65	54	31	23	
≥ 65	98	56	42	
Baseline PSA level (ng/mL)				0.039
≤ 10.0	73	36	37	
10.1–20.0	46	26	20	
> 20.0	33	25	8	
Clinical T stage				0.037
≤ T2	139	76	63	
≥ T3	13	11	2	
Biopsy Gleason score				0.002
≤ 6	43	34	9	
7	66	32	34	
≥ 8	41	19	22	
D'Amico risk group				0.787
Low-risk	18	11	7	
Intermediate-risk	70	38	32	
High-risk	64	38	26	

*Postoperative Pathological Findings*				
Pathological tumor stage				0.212
pT2	95	53	42	
≥ pT3	52	29	23	
pT0	4	4	0	
Pathological lymph node stage				0.153
N0	125	75	50	
N1	11	9	2	
Not stated	16	3	13	
Prostatectomy specimen Gleason score				< 0.001
≤ 6	36	30	6	
7	73	34	39	
≥ 8	33	16	17	
Not stated	10	7	3	
Surgical margin status-no. (%)				0.037
Positive	62 (40.8)	29 (33.3)	33 (50.8)	
Negative	86 (56.6)	55 (63.2)	31 (47.7)	
Not stated	4	3	1	

*Follow-up characteristics*				
Median follow-up months	117.5	173.0	83.0	< 0.001
IQR	81.0–181.0	110.8–209.3	74.8–107.8	
Median months to BCR	27.0	37.0	20.0	0.001
IQR	11.0–63.0	12.0–80.3	8.5–36.5	
Median months to DM	24.0	54.0	12.0	0.101
IQR	13.0–98.5	17.8–144.3	6.0–24.8	
PSA doubling time at BCR, months-no. (%)				0.232
> 9	35 (23.0)	23 (26.4)	12 (18.5)	
≤ 9	62 (40.8)	33 (37.9)	29 (44.6)	
Mortality-no. (%)				
All-cause mortality	21 (13.8)	19 (21.8)	2 (3.1)	< 0.001
PCa-specific mortality	5 (3.3)	5 (5.7)	0	0.049

*Note:* PCa, prostate cancer; y, years.

Abbreviations: BCR, biochemical recurrence; DMs, distant metastases; IQR, interquartile range; ORRP, open radical retropubic prostatectomy; PSA, prostate-specific antigen; RARP, robot-assisted radical prostatectomy.

**Table 2:** Local recurrence and sites of distant metastases.

	All	ORRP	RARP
	(*n* = 152)	(*n* = 87)	(*n* = 65)
Local alone-*n*. (%)	2 (1.3)	1 (1.2)	1 (1.5)
Bones-*n*. (%)	6 (3.9)	3 (3.4)	3 (4.6)
Visceral-*n*. (%)	1 (0.7)	1 (1.2)	0
Bones and LNs and visceral-*n*. (%)	2 (1.3)	2 (2.3)	0

*Note:* LNs, lymph nodes.

Abbreviations: ORRP, open radical retropubic prostatectomy; RARP, robot-assisted radical prostatectomy.

**Table 3:** Cox univariable and multivariable analyses for overall survival in patients with biochemical recurrence after radical prostatectomy.

	Univariable			Multivariable		
	HR	95% CI	*p* value	HR	95% CI	*p* value
Age (y)						
< 65	1			1		
≥ 65	2.440	0.890–6.690	0.083	10.169	0.495–208.882	0.133
Baseline PSA level (ng/mL)						
≤ 10	1			1		
10.1–20.0	1.211	0.405–3.619	0.732	1.146	0.048–27.274	0.933
> 20.0	2.077	0.748–5.764	0.161	13.992	0.592–330.528	0.102
Clinical T stage						
T1	1			1		
T2	0.775	0.296–2.024	0.602	5.463	0.172–173.713	0.336
T3	1.757	0.466–6.630	0.406	56.820	0.501–6448.983	0.094
T4	15.27	1.721–135.481	0.014	324.498	0.715–147246.245	0.064
Biopsy Gleason score						
≤ 6	1			1		
7	2.564	0.831–7.908	0.101	0.672	0.008–53.712	0.859
≥ 8	2.836	0.858–9.376	0.088	0.025	0.009–4.713	0.167
Pathological T stage						
T2	1			1		
≥ T3	1.738	0.706–4.283	0.229	16.801	0.229–1234.181	0.198
Pathological lymph node stage						
N0	1			1		
N1	3.431	1.136–10.361	0.029	0.661	0.002–227.461	0.889
Postoperation Gleason score						
≤ 6	1			1		
7	0.745	0.254–5.385	0.593	0.034	0.021–5.052	0.186
≥ 8	1.566	0.455–5.385	0.477	2.060	0.035–120.139	0.728
Surgical margin status						
Negative	1			1		
Positive	1.295	0.499–3.357	0.595	0.587	0.027–12.811	0.735
Time to BCR (months)	0.987	0.975–0.999	0.034	0.956	0.916–0.997	0.036
PSA doubling time at BCR						
> 9 months	1			1		
≤ 9 months	9.179	1.179–71.472	0.034	6.799	0.187–246.511	0.295

*Note:* 95% CI, 95% confidence interval.

Abbreviations: BCR, biochemical recurrence; HR, hazard ratio; PSA, prostate-specific antigen.

## Data Availability

The data that support the findings of this study are not openly available due to reasons of sensitivity and are available from the corresponding author upon reasonable request.

## Nomenclature

BCR: Biochemical recurrence
BRPC: Biochemically recurrent prostate cancer
ORRP: Open radical retropubic prostatectomy
OS: Overall survival
PCa: Prostate cancer
PSA: Prostate-specific antigen
PSADT: PSA doubling time
RARP: Robot-assisted radical prostatectomy
RP: Radical prostatectomy
TTBR: Time to biochemical recurrence

## Supporting Information

Supporting Table: Cox univariable and multivariable analyses for overall survival in patients with longer TTBR.

Abbreviations: HR, hazard ratio; 95% CI, 95% confidence interval; PSA, prostate-specific antigen; BCR, biochemical recurrence; NE, not estimable.
